# The Sanitation of Armies on Active Service

**Published:** 1906-03-31

**Authors:** 


### The Sanitation of Armies on Active Service.

"It is a commonplace of history, corroborated and confirmed by the experience of almost every campaign, alike in civilised and in uncivilised countries, no less in modern than in ancient times, that disease is a more potent element of warfare than the weapons of the enemy." So writes Dr. Thomas F. Dewar in an admirable essay upon the subject whose title stands at the head of this article.

It is a somewhat surprising circumstance that, in spite of the general recognition accorded by history to the influence of epidemic disease in swaying the fortunes of a campaign, in spite of the immense power for the mitigation of such diseases which the advance of bacteriology and epidemiology has put within our reach, so little serious effort has been expended against the pestilences (particularly typhoid fever and dysentery) which have always stalked in the wake of armies in the field. How indifferent has been our success in preventing in practice diseases which are pre-eminently perventible in theory, is shown by the recent record of the South African War. This campaign extended over a period of thirty-three months. Precise figures are not yet available, but Dr. Dewar considers that it may be said with a close approximation to accuracy that the death-rate from disease was 69 per 1,000 of the total number of troops engaged, while the death-rate from wounds was 12 per 1,000. Excluding the cases of immediately fatal wounds, it appears that the cases admitted to hospital for sickness outnumbered those admitted for wounds by no less a margin than 20 to 1.

If our own experience is not convincing enough, there is no lack of corroboration. Among the French troops engaged in the Crimea 75,000 died of disease, and but 20,000 of wounds; in the French expedition to Madagascar in 1895 only seven men were killed by the enemy, but out of a total of 23,000 engaged, there fell to maladies contracted on service no fewer than 7,500, or almost 33 per cent.

It is clear, therefore, that Dr. Dewar has had no laborious task in demonstrating the urgent need of a more progressive and scientific spirit in the attack upon the problems of service sanitation. This need has been thrown into high relief by the performances of the Japanese in the late war. Upon two occasions only does history tell of a campaign in which the disease death-rate was lower than the wound death-rate. Of these occasions the first was the Franco-Prussian War of 1870-1871, during which the deaths from disease amounted to 18.6 per 1,000, while the deaths from wounds reached 33.7 per 1,000. This, however, is explained by the author of the essay under notice on the score of the brevity of the campaign, the constant movement of the troops, and the favourable character of the season of the year. None of these benign characteristics marked the second and great exception to the rule, the present army of Japan. Throughout a long and arduous campaign, conducted under climatic conditions of quite exceptional rigour, the Japanese are reckoned to have lost by sickness 15,300 men, as against 57,150 killed in battle or subsequently dead of injury.

Much remains to be done before we can hope to vie with the excellence of the military hygiene of the Japanese, and all students of the question may be commended to Dr. Dewar's essay, which embodies a wide personal experience of military service with a practical moderation too rarely seen in specialists. The author is under no delusions as to the proper place of the sanitarian in the hierarchy of the field. In the words of an authority quoted in the text: "sometimes the prevention of disease in the army and the care of the wounded are written about as if they were the objects for which the whole military service existed"; and this unregulated urging of counsels of perfection is likely to do more harm than good. It must be admitted that considerations of hygiene must be subordinated to strategy and tactics, drill and discipline, and an adequate commissariat. But even these exceptions leave to military hygiene a position of commanding importance, for very brief reflection will suffice to establish the immense saving of valuable material which can be effected by a reasonably strict observance of the well-known laws governing the spread of zymotic maladies like dysentery and typhoid fever; and this apart from the economy in labour, money, and appliances postulated by a reduction in the numbers of sick men in the field.

